# Nutritional Status of Cancer Patients During the COVID-19 Pandemic

**DOI:** 10.3390/medicina62081584

**Published:** 2026-08-18

**Authors:** Aydın Aytekin, Ümit Bilge

**Affiliations:** 1 Division of Medical Oncology, Department of Internal Medicine, Faculty of Medicine, Gaziantep University, 27310 Gaziantep, Türkiye; 2 Department of Internal Medicine, Faculty of Medicine, Gaziantep University, 27310 Gaziantep, Türkiye

**Keywords:** COVID-19, malignancy, nutrition, NRS-2002

## Abstract

*Background and Objectives*: The pandemic caused by the SARS-CoV-2 infection has been named coronavirus disease 2019 (COVID-19) by the World Health Organization (WHO). Cancer patients may have been more adversely affected during the COVID-19 Pandemic due to their receipt of immunosuppressive treatments, higher burden of comorbidities, and greater nutritional vulnerability compared to the general population. The aim of our study is to identify potential measures by detecting changes in the nutritional statuses of patients with an oncological diagnosis who presented to our hospital during the COVID-19 pandemic, using the globally accepted NRS-2002 questionnaire. *Materials and Method*: Our study included 324 patients diagnosed with malignancy who applied to the outpatient clinic of Gaziantep University, Şahinbey Training and Research Hospital, Gaziantep/Türkiye, between December 2021 and January 2022 or were hospitalized in the service. Patients included in the study were evaluated in terms of clinical–demographic information and COVID-19-related characteristics using a sociodemographic data form we developed, and then the NRS-2002 questionnaire was administered. *Results*: Our study included 324 patients. Overall, 49.07% (159 patients) of the patients were male, and 50.93% (165 patients) were female. A total of 20.37% of our patients (66 patients) had COVID-19 disease. While those living with their families constituted 96.91% of the patients, at least one of the family members of 36.42% of the patients had COVID-19 disease. While 76.23% (247 patients) of the patients were vaccinated, 23.77% (77 patients) were not vaccinated. When NRS analyses were examined according to the type of malignancy, a statistically significant difference was found between NRS analyses according to the type of diagnosis. When NRS analyses were examined according to the stages of the diseases, it was determined that stage 4 patients were in the risk group in terms of nutrition. When NRS analyses according to age were examined, it was determined that patients over the age of 65 were in the risk group in terms of nutrition. When NRS analyses by gender were examined, it was found that male patients were in the risk group in terms of nutrition. When the NRS analyses were examined according to whether or not radiotherapy was administered, it was determined that the patients who received radiotherapy were in the risk group in terms of nutrition. Contrary to the literature, no statistically significant difference was found according to whether or not chemotherapy was administered. *Conclusions*: According to NRS analysis, no statistically significant differences were found in terms of nutritional status according to COVID-19 transmission and vaccination status. When NRS analyses were examined according to the delay of treatment during the pandemic period, it was found that patients who postponed treatment were in the risk group in terms of nutrition, while patients who did not postpone treatment were mostly in the follow-up group. In multivariable analyses, however, no independent relationship was identified between this parameter and NRS-2002 risk.

## 1. Introduction

The World Health Organization (WHO) declared the COVID-19 outbreak a Public Health Emergency of International Concern (PHEIC) on 30 January 2020. Subsequently, on 11 March 2020, it was characterized as a pandemic due to the alarming levels of spread and severity of the virus, following the detection of cases in 113 countries outside the initial epicenter in China [1]. In a study conducted in Italy, male gender, smoking, obesity, hyperlipidemia, chronic kidney disease, and malignancy were identified as risk factors influencing the prognosis and mortality of COVID-19 [2]. It was emphasized that both older individuals and patients with comorbidities experienced more severe courses of infection. During the COVID-19 pandemic, countries worldwide imposed various restrictions, compelling older populations and individuals with chronic diseases to remain at home and isolated from social environments. Cancer patients, like other individuals with chronic illnesses, underwent home quarantine. This situation led to increased concerns among cancer patients, including anxiety, depression, feelings of loneliness, and fear of death. Furthermore, cancer patients may have been more adversely affected during the COVID-19 Pandemic due to their receipt of immunosuppressive treatments, higher burden of comorbidities, and greater nutritional vulnerability compared to the general population [3,4]. Socioeconomic factors—such as crowded household environments, poor and densely populated working conditions, inadequate hygiene, and insufficient access to protective equipment—also played a significant role in increasing the risk of transmission [3,4]. The objective of this study is to identify the nutritional statuses of oncology patients admitted to Gaziantep University Hospital Oncology Clinic in Gaziantep/Türkiye during the COVID-19 pandemic using globally recognized screening tools and the Nutritional Risk Screening (NRS-2002) questionnaire. Furthermore, this study aims to facilitate the development of strategies and preventive measures targeting the nutritional statuses of cancer patients in the event of potential future pandemics.

## 2. Materials and Methods

### 2.1. Study Design and Patient Selection

A total of 324 patients with a diagnosis of malignancy who either presented to the outpatient clinic or were hospitalized at Gaziantep University Şahinbey Training and Research Hospital, Gaziantep/Türkiye between December 2021 and January 2022 were included in this study. The Clinical data of the patients surveyed were obtained from our electronic system through retrospective scanning. The study was conducted in accordance with the principles of the Declaration of Helsinki and applicable regulations concerning patients’ rights. Ethical approval was obtained from the Ethics Committee of Gaziantep University Faculty of Medicine (decision no. 2021/317; 15 December 2021) before the study was initiated. Patient data were obtained from medical records, the hospital automation system, and the pathology archives. In addition to clinical and demographic information, patients were evaluated using a sociodemographic data form designed to assess whether they postponed hospital appointments during the pandemic, delayed treatment due to fear, had a history of COVID-19 infection, and lived with household members diagnosed with COVID-19, along with their COVID-19 vaccination status. Subsequently, the NRS-2002 screening tool was administered to all patients.

### 2.2. Nutritional Risk Screening (NRS 2002)

The NRS-2002 nutritional screening tool was developed in 2002 by Kondrup and colleagues, with contribution from the Danish Society for Parenteral and Enteral Nutrition [5]. This screening tool is intended to assess individuals’ levels of undernutrition and their risk of malnutrition. The scale initially involves a pre-screening test administered to individuals. In the pre-screening phase, participants are asked whether their body mass index (BMI) is less than 20.5 kg/m^2^, whether they have experienced weight loss in the past three months, whether there has been a reduction in food intake in the previous week, and whether their clinical condition is severe. If at least one of these questions is answered affirmatively, the main screening phase is initiated; if all responses are negative, the pre-screening is repeated at regular intervals.

The main screening component of the scale assesses the degree of nutritional impairment and the severity of the disease. Nutritional impairment is categorized as absent (0 points), mild (1 point), moderate (2 points), or severe (3 points), based on the percentage of weight loss. Disease severity is scored similarly on a scale from 0 to 3. The scores obtained from both components are aggregated. For patients aged 70 years and older, an additional point is added to the calculation as an age-related factor. A total score of 3 or higher indicates the presence of nutritional risk, necessitating the implementation of a nutritional support plan. If the total score remains below 3, the screening process is repeated at regular intervals [6].

Inclusion Criteria: Patients aged ≥ 18 years, diagnosed with solid malignancy, followed up at our institution, and with complete medical records.

Exclusion Criteria: Patients declining participation, patients with incomplete medical documentation, patients managed at external centers, and individuals aged < 18 years.

### 2.3. Statistical Method

Descriptive statistics of the data obtained from the study were presented as mean and standard deviation for numerical variables, and as frequency and percentage for categorical variables. The normality of the variables derived from the scales was assessed using the Shapiro–Wilk test, which indicated that the data were not normally distributed (*p* < 0.05). For the comparison of these variables across categorical groups, the Mann–Whitney U test was employed for categorical variables with two groups, while the Kruskal–Wallis test was used for those with three or more groups. Additionally, Dunn’s test was performed as a post-hoc multiple comparison analysis. Differences between categorical variables were evaluated using Chi-square analysis. Logistic regression analysis was employed for multivariable analyses. Statistical analyses were performed using IBM SPSS Statistics for Windows, Version 22.0 (IBM Corp., Armonk, NY, USA. A *p*-value of <0.05 was considered the threshold for statistical significance.

## 3. Results

A total of 324 patients were included in the study. Of these, 49.07% (*n* = 159) were male, and 50.93% (*n* = 165) were female. The vast majority of patients had health insurance (98.77%). While 267 patients (82.41%) resided in urban areas, 57 patients (17.59%) lived in rural areas. Most patients (49.38%) had completed primary school education. In terms of disease stage, 70.06% of the patients (*n* = 227) were at stage IV, 9.57% (*n* = 31) at stage III, 18.21% (*n* = 57) at stage II, and 2.16% (*n* = 7) at stage I. Among the total cohort, 96.3% of patients received chemotherapy, while 32.72% underwent radiotherapy (Table 1).

Among the patients included in the study, 87.96% (*n* = 285) were married, 8.02% (*n* = 26) were widowed, and 4.01% (*n* = 13) were single. During the pandemic period, 58.64% of the patients (*n* = 190) postponed their outpatient clinic visits, while 32.1% (*n* = 104) delayed receiving chemotherapy. A total of 20.37% of the patients (*n* = 66) had a history of COVID-19 infection. While 96.91% of the patients were living with their families, at least one family member of 36.42% of the patients had been diagnosed with COVID-19. Additionally, 76.23% of the patients (*n* = 247) had been vaccinated, whereas 23.77% (*n* = 77) had not received vaccination (Table 2).

As shown in Table 3, the median age of the patients was 58 (range: 19–83) years. The median body weight was 70 (35–125) kg, the median height was 165 (140–196) cm, and the median body mass index (BMI) was 25.08 (13.84–45.91) kg/m^2^ (Table 3).

Of the patients included in the study, 22.8% (*n* = 74) were followed up for breast cancer, 19.7% (*n* = 64) for colorectal cancer, 17.5% (*n* = 57) for lung cancer, and 15.1% (*n* = 49) for genitourinary system cancers (Table 4).

In our study, patients’ nutritional requirements were assessed based on the NRS-2002 questionnaire. Analysis of NRS-2002 scores according to malignancy type revealed that patients followed for breast cancer were not at nutritional risk. In contrast, patients with gastric, esophageal, and pancreatobiliary malignancies were found to be in the nutritionally at-risk group. A statistically significant difference was observed in NRS-2002 analyses according to diagnosis type (*p* = 0.001). When NRS-2002 analyses were evaluated by disease stage, patients with stage IV disease were found to be in the nutritionally at-risk group, whereas patients with stage III disease were more frequently classified in the follow-up (non-risk) group. Although no statistically significant difference was detected between stages based on NRS-2002 analysis, the result was found to be close to the threshold of statistical significance (*p* = 0.052). When assessing NRS-2002 analyses by age, patients over the age of sixty-five were found to be in the nutritionally at-risk group, whereas patients under sixty-five were predominantly in the follow-up group. A statistically significant difference was observed between age groups according to the NRS-2002 analysis (*p* = 0.001). Furthermore, when evaluating the impact of radiotherapy, patients receiving radiotherapy were identified as being in the nutritionally at-risk group, while those not receiving treatment were more likely to be in the follow-up group. A statistically significant difference was detected between the radiotherapy groups based on the NRS-2002 analysis (*p*= 0.005). Regarding NRS-2002 analyses by gender, male patients were found to be in the nutritionally at-risk group, whereas female patients were more frequently classified in the follow-up group. Furthermore, a statistically significant difference was identified between the gender groups according to the NRS-2002 analysis (*p* = 0.046). When NRS-2002 analyses were evaluated according to treatment delay during the pandemic period, patients who postponed their treatment were found to be in the nutritionally at-risk group, whereas those who did not postpone treatment were more frequently classified in the follow-up group. A statistically significant difference was observed between the groups based on treatment delay (*p*= 0.009). According to NRS-2002 analyses, no statistically significant differences in nutritional risk were identified with respect to chemotherapy status, educational level, history of COVID-19 infection, vaccination status, or postponement of follow-up during the pandemic (while treatment delay itself was significant) (*p* > 0.05). Multivariable analyses (backward likelihood-ratio logistic regression) were performed for the parameters found to be statistically significant, including diagnosis, age, sex, radiotherapy, chemotherapy, and postponement of chemotherapy during the pandemic period. Independent associations were identified between NRS-2002 risk and all parameters except for disease stage and postponement of chemotherapy during the COVID-19 pandemic. No statistically significant association was found between NRS risk and the four COVID-19–related parameters: vaccination status, history of COVID-19 infection, postponement of follow-up visits, and family contact. (Table 5)

## 4. Discussion

With the COVID-19 pandemic, routine practices in oncology clinics had to undergo substantial changes. Even prior to the pandemic, malnutrition was a common issue in oncology, often associated with increased treatment-related toxicity. Impaired nutritional status is linked to poorer response to cancer treatment, reduced quality of life, and worse prognosis. Even in the context of this public health crisis, nutritional support remains a critical component of cancer care [7,8]. During the COVID-19 pandemic, there was a particularly significant and tangible increased risk, especially among oncology patients. Along with the pandemic, disruptions in treatment have emerged in cancer patients, as has been observed in many other chronic diseases. Numerous studies have been conducted to evaluate whether patients avoided hospital admissions to minimize the risk of contracting COVID-19. The literature presents conflicting data regarding this issue. For instance, a study evaluating approximately 4500 patients demonstrated that 90% of the participants did not postpone their outpatient clinic follow-ups due to the pandemic [9]. In another study conducted within the Polish population, it was found that approximately 40% of patients postponed their outpatient follow-up appointments, while 20% delayed their chemotherapy treatment [10].

In our study, during the pandemic period, 58.64% of patients (*n* = 190) postponed their outpatient follow-up visits, while 32.1% (*n* = 104) delayed receiving chemotherapy. It is well established that the risk of malnutrition is higher in gastrointestinal cancers and in tumors at the metastatic stage. Numerous studies have demonstrated that, while the risk of malnutrition does not increase significantly in cancers such as breast and lung cancer, it is significantly elevated in gastrointestinal malignancies, including colorectal, esophageal, gastric, and pancreatic cancers [11,12]. In our study, patients’ nutritional needs were evaluated using the Nutritional Risk Screening (NRS-2002) questionnaire. Analysis of NRS-2002 results according to malignancy type indicated that patients followed for breast cancer were not at nutritional risk. In contrast, patients with gastric, esophageal, and pancreatobiliary malignancies were found to be in the nutritionally at-risk group. A statistically significant difference was observed among NRS-2002 analyses according to diagnosis type (*p* = 0.001). Disease stage has also been identified as a significant factor regarding the risk of malnutrition in numerous studies [12,13]. In our study, however, contrary to the literature, NRS-2002 analyses according to disease stage indicated that patients with stage IV disease were in the nutritionally at-risk group, whereas patients with stage III disease were more frequently classified in the follow-up group. According to the NRS-2002 analysis, no statistically significant difference was observed between stages; however, the result was close to the threshold of statistical significance (*p* = 0.052). The current literature generally reports that gender does not affect the prevalence of malnutrition in cancer patients, whereas the frequency of malnutrition is reported to increase in older patients [12]. In our study, NRS-2002 analyses according to age demonstrated that patients aged 65 years and older were in the nutritionally at-risk group, whereas those younger than 65 years were more frequently classified in the follow-up group. A statistically significant difference was observed between age groups based on the NRS-2002 analysis (*p* = 0.001). In our study, contrary to the literature, NRS-2002 analyses according to gender indicated that male patients were in the nutritionally at-risk group, whereas female patients were more frequently classified in the follow-up group. A statistically significant difference was observed between gender groups based on the NRS-2002 analysis (*p* = 0.046). This finding may be attributed to the higher proportion of male patients with gastrointestinal malignancies. Existing studies have demonstrated that patients receiving radiotherapy and chemotherapy are at high risk for malnutrition [12]. In our study, NRS-2002 analyses based on radiotherapy status demonstrated that patients receiving radiotherapy were in the nutritionally at-risk group, whereas those not receiving radiotherapy were more frequently classified in the follow-up group. A statistically significant difference was observed between the radiotherapy groups (*p* = 0.005). In contrast, and contrary to the literature, no statistically significant difference was found in terms of nutritional risk according to chemotherapy status (*p* > 0.05).

COVID-19 is thought to impose an additional risk of malnutrition in patients with cancer. It has been demonstrated that the hyperinflammation and immunosuppression caused by COVID-19 further increase the risk of malnutrition. In a prospective study of 57 patients conducted by Heng Liu et al., cancer patients who had COVID-19 were found to have a higher risk of malnutrition compared to those who had not, whereas no difference in malnutrition risk was observed among vaccinated patients [14]. In our study, however, NRS-2002 analyses revealed no statistically significant differences in nutritional status regarding history of COVID-19 infection or vaccination status (*p* > 0.05).

There are relatively few studies in the literature evaluating the impact of postponing treatment and/or delaying outpatient follow-up visits on the risk of malnutrition in cancer patients during the pandemic period. While patients undergoing treatment have been shown to have a higher risk of malnutrition, most studies have not demonstrated a significant difference in malnutrition risk with respect to the postponement of follow-up visits [15]. In our study, contrary to the literature, NRS-2002 analyses according to treatment delay during the pandemic demonstrated that patients who postponed their treatment were in the nutritionally at-risk group, whereas those who did not postpone treatment were more frequently classified in the follow-up group. This finding may be attributed, in particular, to the higher proportion of stage IV patients, in whom treatment delay may increase the risk of disease progression and, consequently, the risk of malnutrition. A statistically significant difference was observed between the groups based on treatment delay (*p* =0.009). Although this factor was not identified as an independent risk factor in the multivariable analysis, it may provide guidance for future studies. Treatment delays, particularly in advanced-stage disease, may be associated with disease progression and malnutrition. Consistent with the literature, no statistically significant difference in nutritional status was found according to the postponement of outpatient follow-up during the pandemic period (*p* > 0.05).

## 5. Conclusions

COVID-19 poses multiple additional challenges for oncology patients, a particularly vulnerable patient population. Because the pandemic period necessitated special precautions in many respects and occurred in a context where collective scientific knowledge was limited, studies conducted in this field are of importance for potential future pandemics. In our study, we aimed to emphasize the importance of preventive measures that may be implemented in future pandemic settings by evaluating malnutrition among oncology patients with respect to postponement of outpatient follow-up visits, delay of treatment, and vaccination status—areas for which the current literature remains limited. Given that COVID-19 cases continue to occur and that pandemic-related risk persists, we anticipate that further research in this area will contribute to improving the nutritional status of oncology patients.

As with any study, our study has certain limitations. Due to the study’s cross-sectional, questionnaire-based design, it was not possible to obtain long-term follow-up data or to make direct comparisons with patients treated outside the pandemic period. In addition, the study population was highly heterogeneous, including patients with different diagnoses, cancer stages, and treatment modalities, such as adjuvant, neoadjuvant, and palliative treatments as well as radiotherapy. It was not feasible to obtain a sufficient sample size for analyses restricted to more homogeneous patient groups with similar characteristics. The primary objective of the study was to determine whether the nutritional statuses of patients who had been vaccinated against SARS-CoV-2 and/or had experienced COVID-19 infection were adversely affected. Overall, no clear association was identified between a history of COVID-19 infection and/or vaccination status and nutritional status. In addition to disease stage, factors such as treatment setting, duration of disease, organ involved, treatment modality, and comorbidities could not be evaluated in this study. A larger sample size would have enabled more robust analyses across multiple subgroups.

## Figures and Tables

**Table 1:** Sociodemographic Data-1.

		*n* (%)
Gender	Male	159 (49.07)
	Female	165 (50.93)
Social insurance	Yes	320 (98.77)
	No	4 (1.23)
Place of residence	Urban	267 (82.41)
	Rural	57 (17.59)
Disease Stage	Stage 1	7 (2.16)
	Stage 2	59 (18.21)
	Stage 3	31 (9.57)
	Stage 4	227 (70.06)
Chemotherapy	Yes	312 (96.3)
	No	12 (3.7)
Radiotherapy	Yes	106 (32.72)
	No	218 (67.28)
Educational status	Illiterate	75 (23.15)
	Primary School	160 (49.38)
	Middle School	25 (7.72)
	High School	39 (12.04)
	University	25 (7.72)

**Table 2:** Sociodemographic Data-2.

		*n* (%)
Marital status	Married	285 (87.96)
	Single	13 (4.01)
	Widowed	26 (8.02)
Deferral of outpatient clinic visits during the pandemic	Yes	190 (58.64)
	No	134 (41.36)
Deferral of outpatient chemotherapy during the pandemic	Yes	104 (32.1)
	No	220 (67.9)
History of COVID-19 infection	Yes	66 (20.37)
	No	258 (79.63)
Who does the patient live with?	Living with Family	314 (96.91)
	Living alone	10 (3.09)
Any family history of COVID-19	Yes	118 (36.42)
	No	206 (63.58)
Vaccinated against COVID-19	Yes	247 (76.23)
	No	77 (23.77)

**Table 3:** Sociodemographic Data-3.

	Mean ± SD	Median (Min–Max)
Age	56.19 ± 13.14	58 (19–83)
Weight (Kg)	70.33 ± 13.6	70 (35–125)
Height (Cm)	164.9 ± 8.83	165 (140–196)
BMI (Body Mass Index)	25.96 ± 5.26	25.08 (13.84–45.91)

**Table 4:** Sociodemographic Data-4.

Diagnosis	*n*	%
Breast Cancer	74	22.8
Colorectal Cancer	64	19.7
Lung Cancer	57	17.5
Genitourinary System Cancers	49	15.1
Pancreatic and Biliary Tract Cancers	33	10.1
Stomach and Esophageal Cancers	14	4.32
Head and Neck Cancers	13	4.01
Other Cancers	20	6.17

**Table 5:** NRS 2002 Analyses.

		NRS 2002				*p*
		Risk Group		Follow-Up Group		
		*n*	%	*n*	%	
Diagnosis	Lung Cancer	30	18.87	27	16.36	0.001 *
	Breast Cancer	15	9.43	59	35.76	
	Head and Neck Cancers	5	3.14	8	4.85	
	Stomach and Esophageal Cancers	11	6.92	3	1.82	
	Pancreatic and Biliary Tract Cancers	25	15.72	8	4.85	
	Colorectal Cancers	33	20.75	31	18.79	
	Genitourinary System Cancers	28	17.61	21	12.73	
	Other Cancers	12	7.55	8	4.85	
Stage	Stage 1	2	1.26	5	3.03	0.052
	Stage 2	25	15.72	34	20.61	
	Stage 3	10	6.29	21	12.73	
	Stage 4	122	76.73	105	63.64	
Age	≥65	66	41.51	36	21.82	0.001 *
	<65	93	58.49	129	78.18	
Chemotherapy	Yes	150	94.34	162	98.18	0.067
	No	9	5.66	3	1.82	
Radiotherapy	Yes	64	40.25	42	25.45	0.005 *
	No	95	59.75	123	74.55	
Education Status	Illiterate	45	28.30	30	18.18	0.232
	Primary School	76	47.80	84	50.91	
	Middle School	12	7.55	13	7.88	
	High School	16	10.06	23	13.94	
	University	10	6.29	15	9.09	
History of COVID-19 infection	Yes	32	20.13	34	20.61	0.915
	No	127	79.87	131	79.39	
Vaccinated against COVID-19	Yes	126	79.25	121	73.33	0.211
	No	33	20.75	44	26.67	
Gender	Male	87	54.72	72	43.64	0.046 *
	Female	72	45.28	93	56.36	
Deferral of outpatient chemotherapy during the pandemic	Yes	62	38.99	42	25.45	0.009 *
	No	97	61.01	123	74.55	
Deferral of outpatient clinic visits during the pandemic	Yes	90	56.60	100	60.61	0.465
	No	69	43.40	65	39.39	

* indicate statistical significance (*p* < 0.05).

## Data Availability

The datasets generated and/or analysed during the current study are not publicly available due but are available from the corresponding author on reasonable request.
